# Transcriptomics Analysis of the Toxicological Impact of Enrofloxacin in an Aquatic Environment on the Chinese Mitten Crab (*Eriocheir sinensis*)

**DOI:** 10.3390/ijerph20031836

**Published:** 2023-01-19

**Authors:** Qiaona Wang, Ziling Xu, Ying Wang, Guangming Huo, Xing Zhang, Jianmei Li, Chun Hua, Shengjie Li, Feng Zhou

**Affiliations:** 1 School of Life Science, Nanjing Normal University, Nanjing 210023, China; 2 School of Food Science, Nanjing Xiaozhuang University, Nanjing 211171, China; 3 School of Food Science and Pharmaceutical Engineering, Nanjing Normal University, Nanjing 210023, China

**Keywords:** enrofloxacin, *Eriocheir sinensis*, transcriptome

## Abstract

Enrofloxacin is an important antimicrobial drug that is widely used in aquaculture. Enrofloxacin residues can have negative effects on aquatic environments and animals. The toxicological effects of different concentrations of enrofloxacin residues in cultured water on Chinese mitten crabs (*Eriocheir sinensis*) were compared. A histological analysis of the *E. sinensis* hepatopancreas demonstrated that the hepatopancreas was damaged by the different enrofloxacin residue concentrations. The hepatopancreas transcriptome results revealed that 1245 genes were upregulated and that 1298 genes were downregulated in the low-concentration enrofloxacin residue group. In the high-concentration enrofloxacin residue group, 380 genes were upregulated, and 529 genes were downregulated. The enrofloxacin residues led to differentially expressed genes related to the immune system and metabolic processes in the hepatopancreas of the Chinese mitten crab, such as the genes for alkaline phosphatase, NF-kappa B inhibitor alpha, alpha-amylase, and beta-galactosidase-like. The gene ontology terms “biological process” and “molecular function” were enriched in the carboxylic acid metabolic process, DNA replication, the synthesis of RNA primers, the transmembrane transporter activity, the hydrolase activity, and the oxidoreductase activity. A Kyoto Encyclopedia of Genes and Genomes pathway analysis determined that the immune and metabolic signal transduction pathways were significantly enriched. Furthermore, the nonspecific immune enzyme (alkaline phosphatase) and the metabolic enzyme system played a role in the enrofloxacin metabolism in the *E. sinensis* hepatopancreas. These findings helped us to further understand the basis of the toxicological effects of enrofloxacin residues on river crabs and provided valuable information for the better utilization of enrofloxacin in aquatic water environments.

## 1. Introduction

The Chinese mitten crab (*Eriocheir sinensis*) is a commonly farmed crustacean species typically found in benthic aquatic environments [1]. The Yangtze River Delta is a primary breeding area for crabs in China [2]. Generally, farmers adopt intensive and high-density farming methods to obtain high returns and profits. This breeding practice leads to a gradual deterioration in water quality and increases the incidence of infectious diseases [3]. To prevent disease outbreaks during crab farming, large amounts of antibiotics are used and, as such, are released into the natural water environment.

Enrofloxacin is a third-generation fluoroquinolone antibacterial drug [4,5] which is often used to treat bacterial infections in crab breeding operations in China because of its wide antibacterial spectrum and high potency [6,7]. Although enrofloxacin is approved for aquaculture in China [8], when used in large quantities, the antimicrobial remains in the aquaculture water and sediment accumulates in aquatic animals. Pharmacokinetic studies on enrofloxacin in crustaceans, including the *Eriocheir sinensis* [9,10,11], Atlantic horseshoe crab [12], and giant mud crab [13], have been reported. Enrofloxacin can be biotransformed in vivo into its major metabolite, ciprofloxacin, in crabs. More importantly, Roca et al. reported that quinolone residues are not degraded during processing and that their presence in food poses a risk to human health [14]. The risk of enrofloxacin and ciprofloxacin residues in aquatic products and the aquatic environment has become an important issue that has attracted increasing attention.

Many recent studies on aquaculture animals have focused on the residual characteristics of enrofloxacin and ciprofloxacin; the rate of biotransformation; and the health risks of consuming residual enrofloxacin and ciprofloxacin in *Procambarus clarkia* [15], *Exopalaemon carinicauda* [16], and *Pangasianodon hypophthalmus* [17]. Su et al. reported that enrofloxacin has significant effects on the gene expression of the cytochrome P450 3 (CYP3), glutathione S-transferases (GST), and phosphoenolpyruvate carboxykinase (PEPCK) in crabs [18], which are key molecules that affect the metabolism, immunity, and antitumor activity of organisms [19,20,21,22]. However, there are still few enrofloxacin toxicology reports on Eriocheir sinensis in cultured water.

In this study, we built a microcosm that authentically simulated aquaculture conditions and used it as a research tool to determine the mechanisms underlying the toxicological effects exerted on crabs through exposure to different concentrations of enrofloxacin. In crustaceans, the hepatopancreas has multiple functions, including nutrient absorption and metabolization, the storage of minerals and energy reserves, the synthesis of lipoproteins, the detoxification of heavy metals, and the excretion of uric acid [23]. In addition, the hepatopancreas is an important organ and is the primary site for the synthesis, excretion, and regulation of immune and metabolic molecules [24]. Su et al. demonstrated that enrofloxacin regulated immunity- and metabolism-related gene expression, such as that of CYP3, GST, and PEPCK [18]. Here, we continued to explore the effect of enrofloxacin on the hepatopancreas by using RNA sequencing. Thus, we collected the hepatopancreatic tissue from *E. sinensis* following 10 days of exposure to two different concentrations of enrofloxacin. Histomorphological observations and transcriptome analyses revealed the toxicological effects of enrofloxacin as well as the mechanisms underlying these effects.

We further analyzed the toxicological effects of different concentrations of enrofloxacin on *E. sinensis* using hepatopancreas transcriptome analyses. These results aided in understanding their effect on the immune system and metabolic process disorders of *Eriocheir sinensis* and laid the foundation for further research on *Eriocheir sinensis* by developing an understanding of the defense mechanism essential for maintaining healthy mitten crabs in aquaculture.

## 2. Materials and Methods

### 2.1. Experimental Design and Sampling

To authentically simulate the culture environment of mitten crabs, three Chinese mitten crab culture drums with the same conditions were selected. Each drum had a diameter of 2 m, height of 1.2 m, and water depth of approximately 1 m. Twelve Chinese mitten crabs of similar sizes and weights were placed in each barrel. The amounts of enrofloxacin, 1.875 and 3.750 g, were determined according to the national drug standard, and the enrofloxacin was evenly sprinkled into two different Chinese mitten crab culture drums. After standing for 24 h, the concentration of enrofloxacin in the culture water was 0.63 mg/L in the low-concentration enrofloxacin residue group and 1.25 mg/L in the high-concentration enrofloxacin residue group. After 10 days of sprinkling, crabs were anesthetized on ice and then sampled from the control, low-concentration, and high-concentration enrofloxacin residue groups. The drugs in the aquaculture water were not completely degraded by the time of sampling. The remaining hepatopancreas samples were stored at −20 °C.

### 2.2. Histopathological Analysis of the Hepatopancreas

Hepatopancreatic tissue samples were immediately fixed in 4% paraformaldehyde for 24 h, dehydrated in gradient concentration of ethanol, and embedded in paraffin wax. Using a microtome, 4–5 μm thick sections were obtained and then stained with hematoxylin and eosin (HE). Histopathological changes were observed under a Nikon 50i optical microscope (Nikon Corporation, Tokyo, Japan).

### 2.3. Total RNA Extraction and Sequencing

The transcriptome sequencing and analyses were conducted by Novogene Co., Ltd. (Beijing, China). Total RNA was extracted using a TRIzol^®^ Reagent Kit (Invitrogen, California, USA) according to the manufacturer’s protocol. The RNA quality and quantity were examined using 1% Tris–acetate (TAE) agarose gel electrophoresis. Equal quantities (0.5 μg) of RNA from *E. sinensis* hepatopancreas samples were separately pooled to eliminate sample variation and to create two main samples. The samples were used for RNA-seq library construction using the NEBNext^®^ Ultra™ RNA Library Prep Kit for Illumina^®^ (New England Biolabs, Ipswich, MA, USA) according to the protocol. The AMPure XP system (Beckman Coulter, Beverly, MA, USA) was used to purify library fragments for selecting complementary DNA (cDNA). After the library was constructed, a Qubit2.0 (Thermo Fisher Scientific, Waltham, MA, USA) Fluorometer was used for initial quantification, and the library was diluted to 1.5 ng/µL. The Agilent 2100 BioAnalyzer (Agilent Technologies, Palo Alto, CA, USA) was used to determine the insert size of the library. For ensuring the library quality, quantitative real-time PCR (qRT-PCR) was used to accurately quantify an effective library concentration of higher than 2 nM. Mixed DNA libraries were diluted to 4–5 pM for sequencing using an Illumina NovaSeq 6000 instrument (Illumina Inc, San Diego, CA, USA).

### 2.4. De Novo Transcriptome Assembly

We removed low-quality adapter sequences by filtering raw reads. The resulting clean reads were assembled to produce complete reference sequences using the Trinity program (v2.4.0; min_kmer_cov:3). Longer contigs were assembled until they could not be extended to either side. The unigenes were obtained by removing redundant transcripts. Using BLASTx (2.2.28+; threshold E-value=1 × 10^−5^), the assembled transcripts were aligned with the following National Center for Biotechnology Information protein databases: nonredundant (NR), nucleotide sequence (NT), protein family (PFAM), gene ontology (GO), and protein sequence (Swiss-Prot). The best hits were used for the functional annotation of the unigenes. Blast2GO (b2g4pipe_v2.5, threshold E-value=1 × 10^−6^) was used to obtain and analyze GO annotations for the uniquely assembled transcripts.

### 2.5. Differentially Expressed Gene (DEG) Analysis

DEGs between the control and residue groups were identified using the DESeq package (http://bioconductor.org/packages/release/bioc/html/DESeq.html, accessed on 30 October 2021). An absolute log2-fold change > 1 and FDR < 0.05 were used as thresholds to define DEGs. DEGs were then subjected to GO and Kyoto Encyclopedia of Genes and Genomes (KEGG) analyses.

### 2.6. Enzymatic Analysis

Acid phosphatase (ACP), alkaline phosphatase (AKP), glutathione sulfotransferase (GSH-ST), and acetylcholinesterase (AchE) activities were measured using commercial kits (Nanjing Jiancheng Bioengineering Institute, Nanjing, China) according to the manufacturer’s instructions.

### 2.7. Statistical Analyses

All data were analyzed using SPSS statistics 20 (IBM Inc., Chicago, IL, USA) through one-way analysis of variance (ANOVA), and differences between groups were analyzed using Student’s *t*-test. Statistical significance was set at *p* < 0.05.

## 3. Results

### 3.1. Enrofloxacin Residues Induced Hepatopancreas Injury in E. sinensis

**Figure 1:**
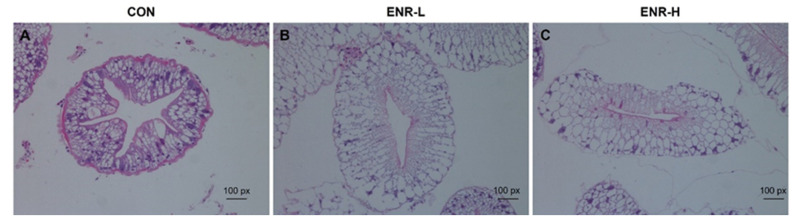
Hematoxylin and eosin staining of *E. sinensis* hepatopancreas specimens following enrofloxacin exposure at different doses. (**A**) CON represents the control group, (**B**) ENR-L represents the low-concentration enrofloxacin residue group, and (**C**) ENR-H represents the high-concentration enrofloxacin residue group.

The degree of hepatopancreatic damage was a direct reflection of the intensity of the toxicity of the external stimuli as observed with the aid of HE staining [25]. In the hepatopancreases of the Chinese mitten crabs in the control group, the basement membranes were complete and clear, and the nuclei were arranged in an orderly manner. The absorbing cells, alveolar cells, fibroblasts, and embryonic cells were clearly distinguished (Figure 1A). However, relative to the control group, in the low-concentration enrofloxacin residue group, the hepatopancreases demonstrated an enlarged space in their lumens and deformed basement membranes; the components in the cells were loosely arranged, the number of nuclei was significantly reduced, and this was accompanied by inflammatory cell infiltration (Figure 1B). In the high-concentration enrofloxacin residue group, the damage to the basement membranes was more severe, the internal structure of the cell membranes was deformed, the internal arrangement of the cells was disordered, the cell structure was lost, secretion in the hepatopancreatic duct cavity was increased, and hepatopancreatic duct atrophy was observed (Figure 1C).

### 3.2. Enrofloxacin Residues Led to Multiple Gene Expression Disorders in the Hepatopancreases of Crabs

**Figure 2:**
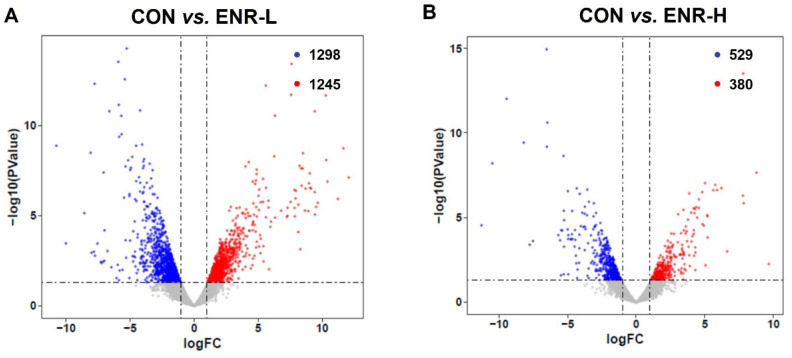
Volcano plot of the differences in the expression profiles of *E. sinensis* samples in the control and residue groups, respectively. CON represents control, (**A**) ENR-L represents low-concentration enrofloxacin residue group, and (**B**) ENR-H represents high-concentration enrofloxacin residue group. The x-axis represents logFC (fold change), while the y-axis represents −log10 (*p*-value). Red represents significantly upregulated genes, blue represents significantly downregulated genes, and each circle represents a single gene.

To elucidate the molecular mechanism underlying the toxicological effects of enrofloxacin on crabs, a de novo assembled transcriptome analysis of the hepatopancreatic samples was performed. The data on the success rate of gene annotation were analyzed in seven databases, including NR, GO, KOG, KO, NT, SwissProt, and PFAM. The annotation success rate in NR was 24,604, accounting for 25.62%; that in GO was 26,538, accounting for 27.64%; that in KOG was 7494, accounting for 7.8%; that in KO was 9251, accounting for 9.63%; that in NT was 17,269, accounting for 17.98%; that in SwissProt was 14,294, accounting for 14.88%; and that in PFAM was 26,542, accounting for 27.64% (Table S1). Through the analysis of the transcriptomic data of the three groups of crab hepatopancreas samples, we obtained DEGs corresponding to the two doses of enrofloxacin. The DEG analysis of the RNA sequence revealed 1245 upregulated and 1298 downregulated genes in the hepatopancreases of the low-concentration enrofloxacin residue group relative to the control group (Figure 2A). Meanwhile, compared with the control group, the DEG analysis demonstrated 380 upregulated and 529 downregulated genes in the hepatopancreases of the high-concentration enrofloxacin residue group (Figure 2B).

### 3.3. GO Analysis of DEGs Found Significant Enrichment of Biological Processes Related to Metabolism Process in Enrofloxacin Residue Groups

**Figure 3:**
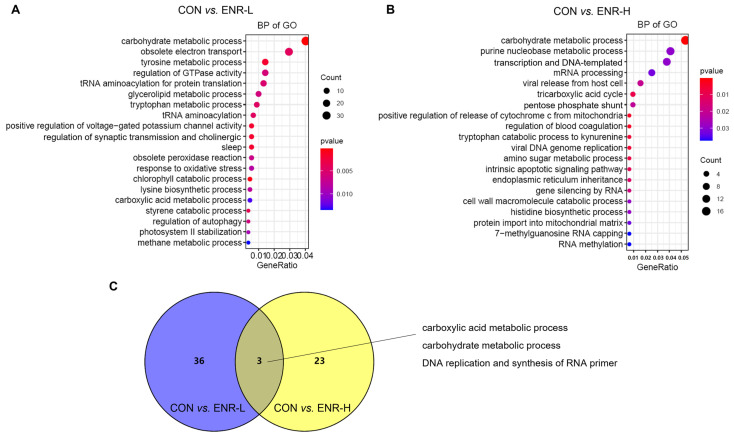
Gene ontology (GO) analysis of the differentially expressed genes (DEGs) in the *E. sinensis* hepatopancreases following different exposure doses of enrofloxacin. Colors represent *p*-values. The x-axis represents the gene ratio. The y-axis represents GO terms involved in biological processes. Venn diagrams showing number of biological processes in *E. sinensis* hepatopancreas samples following (**A**) low and (**B**) high enrofloxacin exposure doses. (**C**) Enrichment of DEGs of the three common biological processes affected by both low (blue) and high (yellow) enrofloxacin exposure doses.

To investigate the function of these DEGs in the hepatopancreases of crabs exposed to enrofloxacin, the 2543 The DEGs corresponding to a low dose of enrofloxacin were entered into the GO database, and the results included the biological processes (BP), cellular components (CC), and molecular function (MF). The DEGs in the low-concentration enrofloxacin residue group, relative to the control group, demonstrated an obvious enrichment of the biological processes chiefly related to the metabolic processes, including the carbohydrate metabolic process (37 DEGs, *p* = 0.000136), tyrosine metabolic process (13 DEGs, *p* = 0.001826), and glycerolipid metabolic process (9 DEGs, *p* = 0.006136; Figure 3A; Table 1). Similarly, the 909 DEGs corresponding to a high dose of enrofloxacin were entered into the GO database, and an enrichment of the biological processes was again evident, including the carbohydrate metabolic process (17 DEGs, *p* = 0.000478), purine nucleobase metabolic process (13 DEGs, *p* = 0.029346), and tricarboxylic acid cycle (3 DEGs, *p* = 0.008759; Figure 3B; Table 1). It is worth noting that the high concentration of enrofloxacin residues led to DNA and RNA damage, and the obvious enrichment in the DEGs involved in translation, DNA-templates (12 DEGs, *p* = 0.029357), mRNA processing (8 DEGs, *p* = 0.032291), and so on (Figure 3B). Metabolic abnormalities were closely related to DNA and RNA damage as well as transcription and translation errors. DNA damage can impair metabolic organ functions and induce tissue inflammation, which disrupts the homeostasis of the systemic metabolism [26]. Moreover, the Venn diagram illustrates that, in the BP of the DEGs, the enrichment between the low- and high-concentration residue groups relative to the control group affected three common biological processes: the carboxylic acid metabolic process, carbohydrate metabolic process, and DNA replication and synthesis of RNA primers (Figure 3C). Therefore, these results illustrated that enrofloxacin residues may affect the hepatopancreatic metabolic processes of *E. sinensis*.

### 3.4. Molecular Function in GO Analysis of DEGs

**Figure 4:**
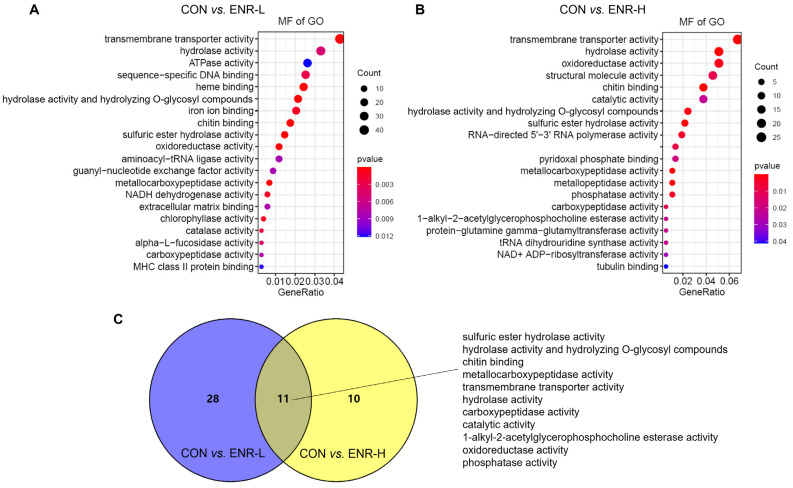
Gene ontology (GO) analysis of the DEGs in *E. sinensis* hepatopancreas samples following different exposure doses of enrofloxacin. Colors represent *p*-values. The x-axis represents the gene ratio. The y-axis represents GO terms involved in molecular functions. Venn diagrams showing number of molecular functions in *E. sinensis* hepatopancreas samples following (**A**) low and (**B**) high exposure doses. (**C**) Enrichment of DEGs of the common molecular functions affected by both low (blue) and high (yellow) enrofloxacin exposure doses.

The molecular function in the GO analysis reflected the biological function that could be affected by the DEGs. Among the molecular functions, the transmembrane transporter activity was most significantly affected in the low-concentration enrofloxacin residue group relative to the control group (44 DEGs, *p* = 0.000256) followed by the hydrolase activity (34 DEGs, *p* = 0.004765), the adenosine triphosphate enzyme (ATPase) activity (27 DEGs, *p* = 0.012104), sequence-specific DNA binding (26 DEGs, *p* = 0.002681), heme binding (25 DEGs, *p* = 2.19 × 10^−5^), the hydrolase activity, hydrolyzing O-glycosyl compounds (22 DEGs, *p* = 4.31 × 10^−6^), iron ion binding (21 DEGs, *p* = 0.001086), chitin binding (18 DEGs, *p* = 2.02 × 10^−5^), the sulfuric ester hydrolase activity (15 DEGs, *p* = 7.41 × 10^−9^), and the aminoacyl-transfer RNA ligase activity (2 DEGs, *p* = 0.036552; Figure 4A). In the high-concentration enrofloxacin residue group, compared with the control, the transmembrane transporter activity was also the most significantly affected (25 DEGs, *p* = 6.42 × 10^−6^) followed by the hydrolase activity (19 DEGs, *p* = 0.000301), the oxidoreductase activity (19 DEGs, *p* = 0.002669), the structural molecule activity (17 DEGs, *p* = 0.009975), chitin binding (14 DEGs, *p* = 2.81 × 10^−8^), the catalytic activity (14 DEGs, *p* = 0.022347), the hydrolase activity and hydrolyzing O-glycosyl compounds (9 DEGs, *p* = 0.048616), and the RNA-directed 5′-3′ RNA polymerase activity (7 DEGs, *p* = 0.004283; Figure 4B). Notably, the molecular functions, including the sulfuric ester hydrolase activity, the hydrolase activity, hydrolyzing O-glycosyl compounds, chitin binding, the metallocarboxypeptidase activity, the transmembrane transporter activity, the catalytic activity, the 1-alkyl-2-acetylglycerophosphocholine esterase activity, the oxidoreductase activity, and the phosphatase activity, occurred repeatedly in both the low- and high-concentration enrofloxacin residue groups (Figure 4C).

### 3.5. Results of KEGG Analysis of DEGs

**Figure 5:**
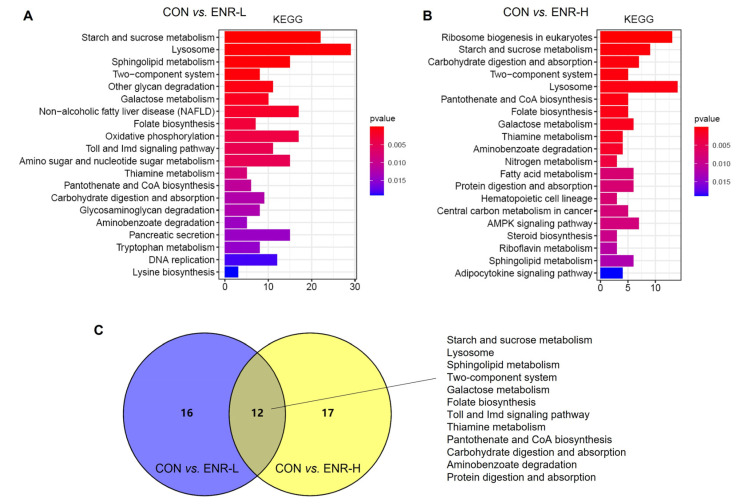
Kyoto Encyclopedia of Genes and Genomes (KEGG) pathway enrichment analysis of DEGs in *E. sinensis* hepatopancreas samples following different exposure doses of enrofloxacin. Colors represent *p*-values. The x-axis represents the number of genes annotated in a KEGG pathway. The y-axis represents the KEGG pathway categories. Venn diagrams showing categories of KEGG pathways in *E. sinensis* hepatopancreas samples following both (**A**) low and (**B**) high exposure doses. (**C**) Enrichment, following KEGG analysis, of DEGs of the system-level biological functions affected by both low (blue) and high (yellow) enrofloxacin exposure doses.

The abundant signaling pathway information in the KEGG database helped elucidate the system-level biological functions, such as the metabolic and inflammatory pathways, oxidative stress, protein modification, and cell death, among others [27]. Relative to the control group, a KEGG enrichment analysis of the DEGs in the low- and high-concentration enrofloxacin residue groups was performed. The KEGG annotation analysis demonstrated that, following the low-dose administration of enrofloxacin, the DEGs were significantly enriched in multiple basic pathways; the abundantly significant pathways were related to the starch and sucrose metabolism, the lysosome metabolism, the sphingolipid metabolism, the two-component system, other glycan degradation, the galactose metabolism, nonalcoholic fatty liver disease, folate biosynthesis, the thiamine metabolism, and the tryptophan metabolism, among others (Figure 5A). In addition, in the high-concentration enrofloxacin administration group, relative to the control group, DEGs were significantly enriched in ribosome biogenesis in eukaryotes; the starch and sucrose metabolism; carbohydrate digestion and absorption; lysosome, pantothenate, and CoA biosynthesis; folate biosynthesis; the galactose metabolism; the thiamine metabolism; the nitrogen metabolism; and the fatty acid metabolism, among others (Figure 5B). The Venn diagram illustrates that 12 pathways were enriched by KEGG following exposure to low or high doses of enrofloxacin, including the starch and sucrose metabolism, the lysosome metabolism, the sphingolipid metabolism, the two-component system, the folate metabolism, the Toll and Imd signaling pathways, the thiamine metabolism, pantothenate and CoA biosynthesis, carbohydrate digestion and absorption, aminobenzoate degradation, and protein digestion and absorption (Figure 5C). Consistent with the GO analysis, the KEGG enrichment analysis also revealed that enrofloxacin exposure may affect the metabolic processes of the crab hepatopancreas.

### 3.6. Enrofloxacin Residues Led to Immune System and Metabolic Process Disorders in the Hepatopancreases of Chinese Mitten Crabs

By analyzing the transcriptomic data of the three groups of crab hepatopancreas samples, we obtained DEGs corresponding to the two doses of enrofloxacin. The results showed that low-concentration enrofloxacin exposure produced 2543 DEGs between the control and experimental groups of which 1245 were upregulated and of which 1298 were downregulated (Figure 2A). In the meantime, high-concentration enrofloxacin exposure yielded a total of 909 DEGs between the control and experimental groups with 380 upregulated and 529 downregulated genes (Figure 2B). Compared with the control group, we found that the DEGs related to the immune system and metabolic processes showed significant changes in both the low- and high-concentration enrofloxacin residue groups. In the immune system, the DEGs, including those for alkaline phosphatase (AKP), dual oxidase 1, and nuclear factor-κB (NF-κB) inhibitor alpha, changed significantly of which NF-κB inhibitor alpha was upregulated in both the low- and high-concentration enrofloxacin residue groups, while the other DEGs were downregulated. In the metabolic processes, the DEGs changed significantly, such as those for venom phosphodiesterase 2-like, beta 1, 4-endoglucanase, alpha-amylase, arylsulfatase A-like, the ecdysteroid receptor (EcR) gene, beta-galactosidase-like, pantothenate kinase 3-like, carboxypeptidase B-like, trypsin-like serine proteinase, chitinase 3, and juvenile hormone esterase-like carboxylesterase 1, and all of them were downregulated in both the low- and high-concentration enrofloxacin residue groups (Table 2). Thus, these results demonstrated that immune system and metabolic process disorders may be key factors in *E. sinensis* hepatopancreatic damage.

### 3.7. Immune Responses and Metabolic Enzymatic Activities following Exposure to Enrofloxacin Residues

**Figure 6:**
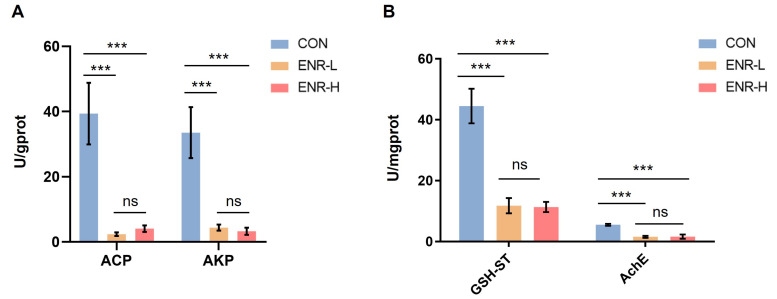
Effects of different enrofloxacin residues on enzyme activity in the hepatopancreases of *E. sinensis* specimens. CON represents the control group, ENR-L represents the low-concentration enrofloxacin residue group, and ENR-H represents the high-concentration enrofloxacin residue group. *** *p* < 0.001. (**A**) Pollution impact indicators, acid phosphatase (ACP) and alkaline phosphatase (AKP), indicating the impact of low- and high-concentration enrofloxacin residues on the immune defense of *E. sinensis*. (**B**) Metabolic process indicators, glutathione sulfotransferase (GSH-ST) and acetylcholinesterase (AchE), indicating the impact of low- and high-concentration enrofloxacin residues on metabolic processes of the liver and pancreas in *E. sinensis*. The meaning of “ns” in figure is “no significance”.

Our analysis revealed that damage to the *E. sinensis* hepatopancreas was primarily associated with immune responses and metabolic processes. By analyzing DEGs, we found that enrofloxacin residues led to immune system and metabolic process disorders in the hepatopancreases of Chinese mitten crabs. The widely used indicators ACP and AKP are potential indicators for evaluating the impact of pollutants on the immune defense of biological organisms [28,29]. The other important indicators GSH-ST and AchE are indicators of the metabolic processes of the liver and hepatopancreas in animals [30,31]. Therefore, we selected those genes from the target DEGs for the qPCR analyses. The results demonstrated that the enzymatic activities of ACP and AKP in the hepatopancreases of the specimens were significantly decreased in both the low- and high-concentration enrofloxacin residue groups (Figure 6A). Notably, the results demonstrated that the enzymatic activities of GSH-ST and AchE were also significantly decreased in both the low- and high-concentration enrofloxacin residue groups (Figure 6B).

## 4. Discussion

Our results indicated that exposure to low or high doses of enrofloxacin resulted in hepatopancreatic damage in *E. sinensis*. The RNA sequencing results demonstrated that enrofloxacin-induced hepatopancreatic damage was closely related to disorders of the metabolic processes and the immune system.

Enrofloxacin, a third-generation fluoroquinolone antibacterial drug, is commonly used to treat bacterial infections in crab breeding; however, the side effects of this antimicrobial treatment deserve attention. For example, the expression level of key genes and enzymes in the hepatopancreases of the *E. sinensis* specimens treated with enrofloxacin were disturbed [18]. The enrofloxacin biotransformation product, ciprofloxacin, at low and high doses affected the expression of many genes in the hepatopancreases of the *E. sinensis* specimens, and these were primarily enriched in the metabolic processes and the immune system.

The metabolism involves a series of reactions that occur in living cells to sustain life [32]. Metabolic disorders can lead to the occurrence and development of various disorders and diseases in organisms that seriously affect the quality of life of these organisms and that may even threaten their lives. In addition, the metabolic disorders of crabs seriously affect the yield and environment of the aquaculture [33,34,35]. In eukaryotes, the metabolic processes are involved in various interconnected cellular pathways, molecular signaling pathways, and metabolic materials and products [36,37,38]. At the molecular level, metabolic changes depend on the configuration of the metabolic pathways, which are regulated by key metabolic enzymes, transcription factors, protein modifications, and the metabolite clearance status; many of these pathways are closely related to the mitochondrial and lysosomal digestive functions [39,40]. In this study, we determined that enrofloxacin exposure affected the metabolism of many substances, including carbohydrates, lipids, starch, and sucrose, as determined by the GO and KEGG enrichment analyses. Specifically, the metabolic processes, including the metabolism of carbohydrates, tyrosine, glycerolipids, tryptophan, methane, purine nucleobases, and amino sugars, were significantly altered following enrofloxacin exposure. Moreover, the functions of the mitochondria and lysosomes were also affected by enrofloxacin. Significant changes in the enrichment results relative to the biological processes (the response to oxidative stress, tricarboxylic acid cycle, and protein import into the mitochondrial matrix) and molecular functions of the GO analysis (the ATPase activity, NADH dehydrogenase activity, and oxidoreductase activity) and KEGG analysis (the lysosome metabolism, oxidative phosphorylation, pantothenate and CoA biosynthesis, and the fatty acid metabolism) were all involved in the resultant damage of the mitochondrial and lysosomal functions following enrofloxacin exposure.

The metabolic pathways are well recognized as important regulators of immune differentiation and activation [41,42,43]. Chen et al. reported that Ophiopogon japonicus increased the immune response in *E. sinensis*, inhibited the proliferation of the white spot syndrome virus (WSSV), and improved the survival of WSSV-challenged crabs [44]. Abamectin insecticides and anthelmintics inflict oxidative damage on aquatic animals and impair the immune defenses, which may further cause a sharp drop in the hemocyte counts in *E. sinensis* [29]. Therefore, immune homeostasis is crucial for *E. sinensis* survival. We determined that enrofloxacin exposure affected the biological processes, including the response to oxidative stress, regulation of autophagy, and viral release from the host cells, all of which were related to the homeostasis of the immune system [45,46,47,48,49]. Moreover, some DEGs related to the immune system showed significant changes in expression, including those of AKP, dual oxidase 1, and NF-κB inhibitor alpha, in the hepatopancreases of the *E. sinensis* specimens.

The widely used indicators ACP and AKP are potential indicators for evaluating the impact of pollutants on the immune defense of biological organisms [28]. Oxidative stress markers, GSH-ST, and AchE are important indicators that affect the metabolic processes of the liver and hepatopancreas in animals [50]. Overall, the ACP, AKP, GSH-ST, and AchE levels were clearly decreased following enrofloxacin exposure in the hepatopancreases of the *E. sinensis* specimens, confirming that enrofloxacin exposure affects the metabolism and immune response in the hepatopancreas of *E. sinensis*. However, the mechanisms by which enrofloxacin affects the metabolism and the immune system remain unclear and require further investigation.

## 5. Conclusions

In conclusion, this study not only provided novel evidence for the toxicological effects exerted on *E. sinensis* following enrofloxacin exposure but also helped elucidate the possible mechanisms underlying this toxicity and the corresponding cellular pathways that were activated, which notably involved the metabolic processes and immune responses.

## Figures and Tables

**Table 1:** The top 20 biological processes (BP) in gene ontology (GO) analysis of differentially expressed genes (DEGs).

CON ^1^ vs. ENR-L ^2^			CON vs. ENR-H ^3^		
Term	Count	*p* Value	Term	Count	*p* Value
carbohydrate metabolic process	37	0.000136285	carbohydrate metabolic process	17	0.000478166
obsolete electron transport	27	0.004245120	purine nucleobase metabolic process	13	0.029346042
tyrosine metabolic process	13	0.001825573	transcription and DNA-templated	12	0.029356640
regulation of GTPase activity	13	0.005738756	mRNA processing	8	0.032290906
tRNA aminoacylation for protein translation	12	0.005255766	viral release from host cell	5	0.020254351
glycerolipid metabolic process	9	0.006135912	tricarboxylic acid cycle	3	0.008758657
tryptophan metabolic process	8	0.004595175	pentose phosphate shunt	3	0.021326841
tRNA aminoacylation	6	0.004389519	regulation of blood coagulation	2	0.004063707
positive regulation of voltage-gated potassium channel activity	5	0.001854393	positive regulation of release of cytochrome c from mitochondria	2	0.004063707
regulation of synaptic transmission and cholinergic	5	0.001854393	tryptophan catabolic process to kynurenine	2	0.006012747
sleep	5	0.001854393	viral DNA genome replication	2	0.006012747
obsolete peroxidase reaction	5	0.006743223	amino sugar metabolic process	2	0.008303677
response to oxidative stress	5	0.008306801	intrinsic apoptotic signaling pathway	2	0.010921666
chlorophyll catabolic process	4	0.000717921	endoplasmic reticulum inheritance	2	0.013852347
lysine biosynthetic process	4	0.007702752	gene silencing by RNA	2	0.017081801
carboxylic acid metabolic process	4	0.012737180	cell wall macromolecule catabolic process	2	0.028430117
styrene catabolic process	3	0.003663577	histidine biosynthetic process	2	0.028430117
regulation of autophagy	3	0.006128731	protein import into mitochondrial matrix	2	0.032724060
photosystem II stabilization	3	0.009375217	7-methylguanosine RNA capping	2	0.037253517
methane metabolic process	3	0.013447069	RNA methylation	2	0.037253517

^1^ Control group, ^2^ low-concentration enrofloxacin residue group, and ^3^ high-concentration enrofloxacin residue group.

**Table 2:** Summary of DEGs related to immune system and metabolic processes in the *E. sinensis* transcriptome.

	CON ^1^ vs. ENR-L ^2^		CON vs. ENR-H ^3^	
	logFC	*p* Value	logFC	*p* Value
Immune system				
alkaline phosphatase	−5.909012989	2.99 × 10^−14^	−1.36058962	3.84 × 10^−2^
dual oxidase 1	−3.065045953	3.06 × 10^−3^	−2.19026803	1.42 × 10^−2^
NF-kappa B inhibitor alpha	1.680036355	1.13 × 10^−2^	1.668237232	2.98 × 10^−2^
metabolic process				
venom phosphodiesterase 2-like	−2.154713643	1.46 × 10^−4^	−1.70584579	5.71 × 10^−3^
beta 1,4-endoglucanase	−4.572645684	6.59 × 10^−3^	−2.81828784	1.40 × 10^−2^
alpha-amylase	−1.803435554	1.97 × 10^−2^	−1.50174728	1.70 × 10^−2^
arylsulfatase A-like	−3.900096603	7.40 × 10^−9^	−1.42018217	1.51 × 10^−2^
ecdysteroid receptor (EcR) gene	−2.393264135	1.87 × 10^−5^	−2.0989689	2.25 × 10^−4^
beta-galactosidase-like	−2.452835189	4.92 × 10^−5^	−1.93785202	8.92 × 10^−4^
pantothenate kinase 3-like	−2.018241358	7.46 × 10^−3^	−1.51740929	3.45 × 10^−2^
carboxypeptidase B-like	−3.554127549	1.86 × 10^−4^	−3.08832125	2.19 × 10^−4^
trypsin-like serine proteinase	−4.213806456	4.03 × 10^−3^	−3.9272743	5.16 × 10^−6^
chitinase 3	−2.837019698	2.05 × 10^−3^	−1.33310288	4.96 × 10^−2^
juvenile hormone esterase-like carboxylesterase 1	−2.794416003	4.66 × 10^−2^	−1.6048277	1.09 × 10^−2^

^1^ Control group, ^2^ low-concentration enrofloxacin residue group, and ^3^ high-concentration enrofloxacin residue group.

## Data Availability

The data sets used and analyzed during the current study are available from the corresponding author upon reasonable request.

## Supplementary Materials

The following supporting information can be downloaded at: https://www.mdpi.com/article/10.3390/ijerph20031836/s1, Table S1: Statistics of gene annotation success rate.
