# Supplementary material for: Transcriptomics Analysis of the Toxicological Impact of Enrofloxacin in an Aquatic Environment on the Chinese Mitten Crab (Eriocheir sinensis)
Source: Int J Environ Res Public Health. 2023 Jan 19;20(3):1836. doi: 10.3390/ijerph20031836 (PMC9915228; doi:10.3390/ijerph20031836)
Supplement: Supplementary file 1 [file ijerph-20-01836-s001.zip › ijerph-2010625-supplementary.pdf]

Database	Number of Unigenes	Percentage
Annotated in NR	24604	25.62
Annotated in NT	17269	17.98
Annotated in KO	9251	9.63
Annotated in SwissProt	14294	14.88
Annotated in PFAM	26542	27.64
Annotated in GO	26538	27.64
Annotated in KOG	7494	7.8
Annotated in all Databases	2785	2.9
Annotated in at least one Database	44144	45.98
Total Unigenes	96003	100

Table S1. Statistics of gene annotation success rate. NR, NCBI non-redundant protein sequences; NT, NCBI nucleotide sequences; KO, kyoto encyclopedia of genes and genomes orthology; PFAM, Protein family; GO, Gene Ontology; KOG, clusters of orthologous groups for eukaryotic complete genomes.
